# Downscaled ERA5 Land addresses agrometeorological data scarcity in North African basins

**DOI:** 10.1038/s41598-025-20552-2

**Published:** 2025-11-04

**Authors:** Youness Ouassanouan, Vincent Simonneaux, Mohamed Hakim Kharrou, Younes Fakir, Mohamed Wassim Baba, Bouchra Ait Hssaine, Chouaib El Hachimi, Laura Sourp, Abdelghani Chehbouni

**Affiliations:** 1 Center for Remote Sensing Applications (CRSA), Mohammed VI Polytechnic University (UM6P), 43150 Ben Guerir, Morocco; 2 CNES/CNRS/INRAE/IRD/UPS, Centre d’Etudes Spatiales de la Biosphère (CESBIO), Université de Toulouse, 31400 Toulouse, France; 3 International Water Research Institute (IWRI), Mohammed VI Polytechnic University (UM6P), 43150 Ben Guerir, Morocco; 4 Department of Geology, Faculty of Sciences–Semlalia, Cadi Ayyad University, 40001 Marrakech, Morocco

**Keywords:** ERA5_Land, Downscaling, MicroMet, Reference evapotranspiration, Data-scarce basins, North Africa, Atmospheric science, Climate change, Hydrology

## Abstract

A reliable estimate of reference evapotranspiration (ET0) requires several meteorological inputs, which may be unavailable in regions with limited data availability. In this regard, this study aimed to address the following objectives: First, to evaluate the effectiveness of ERA5_Land (ERA5_L hereafter) weather forecasts in providing daily agrometeorological variables for the period 2003–2021 at 10 study sites distributed over both plain and mountainous areas in a North African basin. Second, to investigate whether downscaling the ERA5_L data (10 km) to station scale (250 m) using a quasi-physical based model, MicroMet, could improve the reliability of the meteorological variables. Third, to compare the performance of the original ERA5_L reanalysis data and the disaggregated ERA5_L data (MicroMet) as potential sources for accurate estimation of ET0 on a daily time scale. Finally, to assess the long-term spatiotemporal changes in ET0 across the Tensift basin over the period 1950–2021, and the influence of climate variables and topography on ET0 variability. The findings of the study revealed that the original ERA5_L estimates of air temperature (Tair) were the most accurate among the studied variables, followed by solar radiation (Rs), relative humidity (RH), and wind speed (u2). When considering the disaggregated daily ERA5_L data, Tair exhibited the highest performance, followed by RH, u2, and Rs. Tair and especially u2 demonstrated an improvement across the plain and mountainous sites. However, Rs was generally degraded after MicroMet. Then, a comparison was conducted between daily ET0 obtained considering both datasets and show similar correlations between ground and simulated data but with an overestimation of ET0 after MicroMet. Finally, the retrospective analysis of ET0 showed three main phases with a decrease of ET0 between 1950 and 1970, a nearly steady period during 1970–2000, and a significant increase from 2000 to 2021. This study provides a comprehensive insight on the potential and limitations of ERA5_L products in arid North African regarding irrigation and water management under climate variability.

## Introduction

Water management has emerged as a prominent global challenge in the last decades, as numerous regions worldwide are anticipated to face difficulties in meeting the growing demand for freshwater in the upcoming years^1^. Considering that the agricultural sector is responsible for more than two-thirds of global water withdrawals^2^, it is of paramount importance to ensure effective agricultural water management. This includes especially the need to estimate crop water consumption returned to the atmosphere as evapotranspiration (ET). In this aim, several approaches of crop water ET estimates rely on the assessment of a meteorological variable named reference evapotranspiration (ET0), this later which represents ET of a well-watered large, uniform, and actively growing grass cover of 0.12 m height with an albedo of 0.23^3^. The concept of ET0 has been promoted by the Food and Agriculture Organization (FAO), which has standardized the Penman–Monteith (PM) equation (FAO56-PM) as the recommended method for its estimation^4^ using as input four meteorological variables, namely solar radiation (Rs), relative humidity (RH), air temperature (Tair) and wind speed (u2).

Estimating ET0 is useful for assessing crop water demand^5^, water withdrawal estimation^6^, and drought monitoring^7^ and irrigation scheduling^8–11^. It thus contributes to improve agricultural productivity and food security^12,13^. Since ET0 is exclusively influenced by climatic factors, the direct impact of climate change and climate variability on crop water consumption can be assessed. However, despite its effectiveness in estimating ET0, the FAO56-PM method faces limitations due to its extensive data requirements, which have hindered its global applicability, especially in data-scarce regions^14^. Indeed, the four meteorological variables required are not always available in situ or of insufficient density or quality^15^. Although few weather stations may be used, it remains a challenge, especially in heterogeneous landscapes like mountains. Therefore, alternative approaches were developed to provide spatially distributed meteorological data. These include the use of data interpolation methods to derive estimates for specific areas of interest^16–18^. However, the application of geostatistical methods for ET0 estimation requires a densely distributed network of weather stations with precise and high-quality measurements, which is often not available in semiarid and arid regions, especially in complex areas of developing countries in North Africa^19,20^.

Recently, atmospheric reanalysis data have received considerable attention due to their ability to provide consistent global time series of several meteorological parameters over several decades. These freely available gridded datasets cover a wide range of spatiotemporal resolutions and are therefore suitable to substitute observations in regions with limited data availability. In particular, the Modern-Era Retrospective Analysis for Research and Applications (MERRA)^21^ and MERRA2^22^ products generated by the National Aeronautics and Space Administration (NASA), ERA-Interim^23^, ERA5^24^ and ERA5_Land^25^ developed by the European Center for Medium-Range Weather Forecasts (ECMWF) are considered state-of-the-art global meteorological reanalysis datasets. Therefore, it is necessary to disaggregate the required variables at the station level to account for local variations and to consider the effects of topography on these variables^26–28^. Previous studies conducted by^27^ in the Moroccan High Atlas Mountains have shown that the resolution of climate variables should not exceed 500 m to accurately capture the energy budget variability. To overcome this challenge, a physical-based model called MicroMet^29^ was used to downscale the reanalysis data at 250 m resolution.

The objective of our study was, firstly, to evaluate the accuracy of the ERA5_L meteorological variables used for calculating ET0. We compared these variables against measurements from ten ground stations located in both plain and mountainous areas within a North African basin between 2003 and 2021. Secondly, we assessed the potential of disaggregating ERA5_L variables using the MicroMet model^29^. We evaluated the accuracy of ET0 estimates based on both the original and disaggregated ERA5_L reanalysis products by comparing them with ground-based ET0 measurements. Lastly, we discussed the spatiotemporal evolution and the influencing factors of ET0 trends across various climatic and topographic factors in the Tensift basin from the period between 1950 and 2021.

## Methodology

### Study area and data processing

**Fig. 1:**
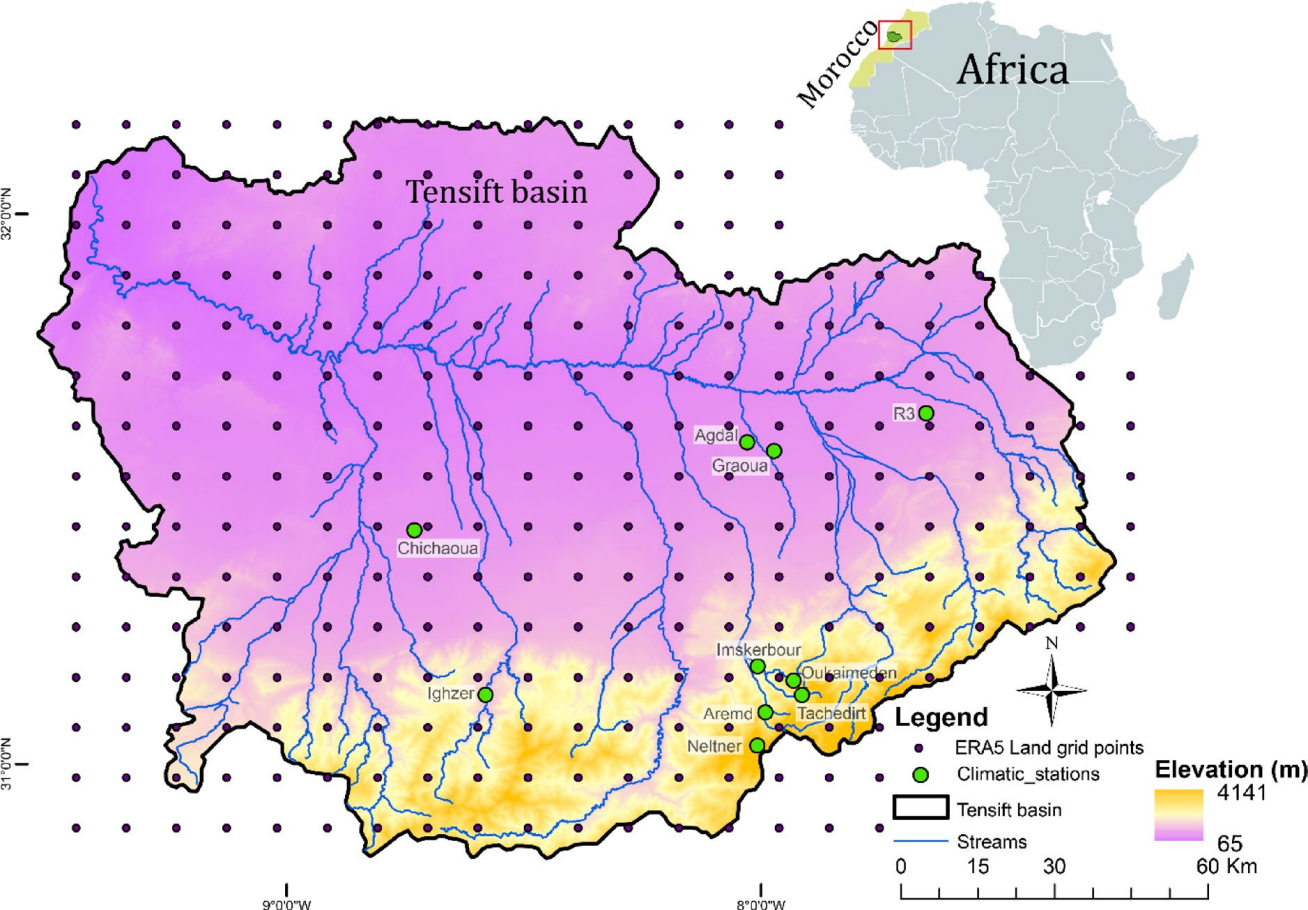
Study site and location of the weather stations.

The study area is the Tensift basin, which extends over an area of 30,000 Km2 between latitudes 30.75–32.40° N and longitudes 7.05–9.9° W (Fig. 1). The Tensift basin in Morocco which is characterized by its topographic variability, with altitudes ranging from 1000 to 4167 m in the High Atlas Mountains and 200 m to 1000 m in the Haouz plain around Marrakech. The basin has a Mediterranean climate with high spatial climatic variability; the High Atlas Mountains have a sub-humid climate, while the plains have a semi-arid to arid climate^30^.

In Morocco, despite decades-long collection of weather data since the 1960’s, achieved by the meteorological department and the watershed agencies, the data remain quite scarce at the basin scale. The Tensift basin stands out as one of the most instrumented basins in Morocco with a network of automatic weather stations (AWSs) installed starting 2002^31,32^. Yet, long-term ground data limitation is a significant challenge, This issue actually was the main motivation of our study, to explore alternative approaches for filling these data gaps. Currently, this network offers a comprehensive collection of meteorological data providing a spatial representation of climate variability from the Haouz Plain to the High-Atlas Mountains with a high temporal resolution of 30 min. For this study, we used data from 10 stations (Fig. 1, Table 1) measuring air temperature, relative humidity, solar radiation, and wind speed.

**Table 1:** AWSs name, coordinates, altitude, and the recording period.

Station	Latitude (degrees)	Longitude (degrees)	Elevation (m)	Period
Agdal	31° 60′ 11″ N	7° 97′ 38″ W	506	2005–2021
Chichaoua	31° 43′ 01″ N	8° 65′ 32″ W	517	2003–2021
Graoua	31° 58′ 68″ N	7° 92′ 07″ W	523	2003–2021
R3	31°66′74″N	7° 59′ 57″ W	593	2013–2021
Ighzer	31° 14′ 42″ N	8° 49′ 71″ W	1035	2018–2021
Imsker	31° 20′ 73″ N	7° 94′ 10″ W	1404	2007–2021
Armed	31° 60′ 11″ N	7° 92′ 07″ W	2050	2002–2021
Tachedirt	31° 15′ 86″ N	7° 84′ 88″ W	2343	2007–2021
Neltner	31° 06′ 74″ N	7° 93′ 77″ W	3207	2007–2021
Oukaimeden	31° 12′ 42″ N	7° 86′ 28″ W	3230	2009–2021

### Data pre-processing and daily ET0 calculation

The FAO56-PM equation was used to calculate the daily ET0, using (1) the original resolution ERA5_L variables (10 km) and (2) the downscaled variables (250 m). ERA5_L data were obtained at hourly resolution, then aggregated to calculate daily averages. In order to compare the ERA5_L data with the in-situ measurements, we have compared the station data with the nearest ERA5_L grid point.

1 $$\text{ET}0=\frac{0.408* \Delta *\left(Rn-G\right)+ \gamma * \frac{900}{\left(Tair+273\right)}* u2*(ea-es)}{\Delta +\upgamma (1+0.34*\text{u}2)}$$

where Rn (W/m^2^) is the net radiation and G (W/m^2^) is the soil heat flux (at a daily time step). Ta is the mean daily Tair (°C), $\Delta$ is the slope of the saturation vapor pressure curve at Tair (kPa/°C), γ is the psychometric constant (kPa/°C), es is the saturation vapor pressure at Tair (kPa), ea is the average daily actual vapor pressure (kPa) and u2 is the average daily wind speed at 2 m height (m/s).

The slope of saturation vapor pressure curve (∆) is derived from Tair as:

2 $$\Delta = \frac{{4089*{\text{es}}}}{{\left( {{\text{ Tair }} + { }237.3} \right)^{2} }}$$

The psychometric constant (γ) is given by:

3 $$\gamma = \frac{{{\text{C}}_{{\text{p}}} *{\text{P}}}}{{\varepsilon *{\uplambda }}}$$

With P as the atmospheric pressure, Cp is the air’s specific heat at constant pressure based on the ideal gas law with a value of 1.013 × 10^−3^ MJ kg^−1^/°C, ε is the ratio of the molecular weight of water vapor to that of dry air, or 0.622, and λ is the latent heat of vaporization.

The net radiation, Rn, is estimated from net solar radiation (Rs) and net thermal radiation (Rt) as:

4 $$Rn=Rs-Rt$$

G is considered to be 10% of Rn during the day and 50% during the night^4^.

5 $$\text{G}day = Rn * 0.1$$

6 $$\text{G}night=Rn*0.5$$

The ea and es were computed using the average Tair and dew point temperature Tdew derived on 24 h basis as inputs using the formula proposed by^4^, which was also recently used in the study by^33^.

7 $$es=0.6108 exp\left(\frac{17.27*\text{ Tair}}{\text{ Tair }+ 237.3}\right)$$

8 $$ea=0.6108 exp\left(\frac{17.27*\text{ Tdew}}{\text{ Tdew }+ 237.3}\right)$$

Then, the relative humidity is calculated as follow:

9 $$RH=100\left(\frac{\text{ea}}{\text{ es}}\right)$$

The wind speed is provided in ERA5_L by its eastward (u) and northward (v) components at 10 m.

10 $$u2=uz\left(\frac{4.87}{\text{ln}\left(67.8z-5.42\right)}\right)$$

With u2 representing wind speed at 2 m, uz is the wind speed at z height above the surface (z = 10 m in ERA5_L) derived from $uz=\sqrt{{u}^{2}+{v}^{2}}$ , for which wind speed data are available.

### ERA5_L data disaggregation

MicroMet is a quasi-physically based model developed by^29^. This model is designed to generate high-resolution weather data required to run terrestrial models across different landscapes. The MicroMet model is based on the relationships between climatic variables and the surrounding landscapes, mainly topography, to distribute these variables for any given landscape in a physically based and efficient computation manner. To that end, MicroMet applies two main adjustments to these climatic data: firstly, a spatial interpolation of all available variables at a given time over the domain; secondly, it performs physical submodels for each MicroMet variable to enhance parameter realism at a given location in time and space. The MicroMet model distributes the main meteorological variables needed to run most terrestrial models, including Tair, RH, Rs, u2, wind direction, incoming longwave radiation, surface pressure, and precipitation. For spatial interpolation, MicroMet employs the Barnes objective analysis scheme for station or horizontal interpolation^34–36^. The Barnes analysis involves interpolating data from an irregularly distributed network of stations to a regular grid by applying a Gaussian distance-dependent weighting function.

In this study, we used the hourly ERA5_L grid points as input data to run MicroMet. In the case of air temperature, a lapse rate correction is applied; for RH, as this is a non-linear function of altitude, the relative linear Tdew value is used for elevation adjustment, then converted to RH using es and ea. Regarding u2, the u and v components are interpolated independently using Barnes objective analysis scheme^34^. The output values are then converted to wind speed and direction; these values are modified using a topographically driven wind model to adjust the speed and direction according to topographic slope and curvature relationships^37^. For Rs the model uses equations that use the model time to calculate the solar radiation for that specific time and account also for the influence of cloud cover, topographic slope, direct and diffuse solar radiation, and aspect on incoming solar radiation. A study by Baba et al. (2019) evaluated the influence of spatial resolution on MicroMet outputs using multiple resolutions (8 m, 30 m, 90 m, 250 m, 500 m, and 1000 m). Their results showed that the differences in the Heidke Skill Score (HSS) between models remain small from 8 to 250 m. However, the median HSS values decrease at 500 m and drop further at 1000 m, indicating that these coarser resolutions are insufficient to capture spatial variability. Based on this analysis, we selected 250 m as an optimal resolution, balancing reliable performance with reasonable computational demands. A more complete description of the MicroMet model and its application can be found in^27–29,38,39^.

### Trend analysis

For this purpose, we have used the most widely employed non-parametric tests, the Mann–Kendall (MK, hereafter) statistical test^40,41^ and the Sen Slope estimator^42^ were applied to the ET0 time series for the period 1950 to 2021. In addition, the Pettitt test^43^ was employed to identify change points and assess data homogeneity. The latter is also a nonparametric test, widely used in hydro‑meteorological studies for detecting abrupt changes in the central trend of a time series without requiring prior knowledge of the change point’s occurrence^44–46^.

## Results

### Agro-meteorological variable-by-variable comparisons

#### Air temperature (Tair)

##### Tair ERA5_L Vs in-situ measurements

**Fig. 2:**
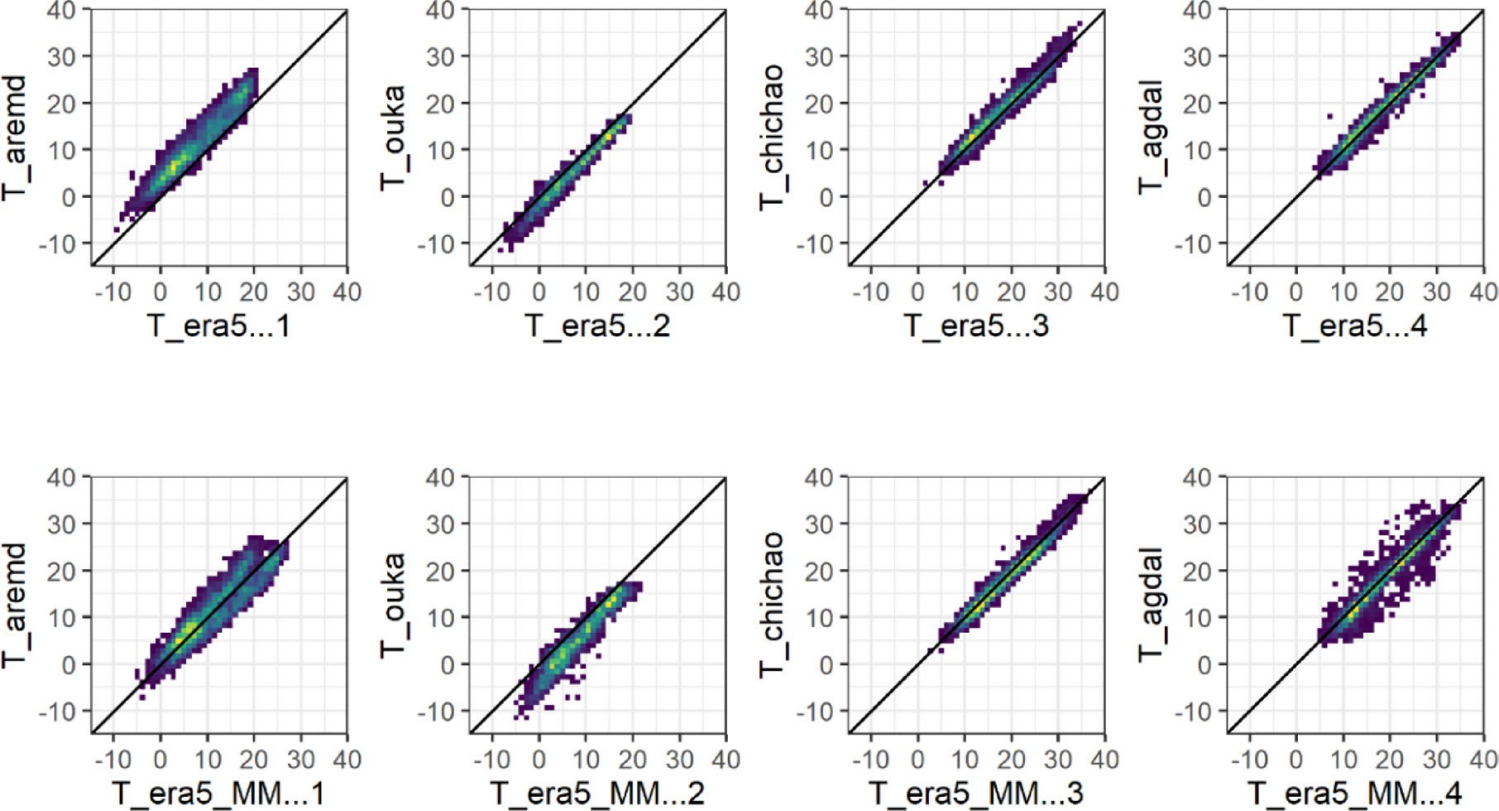
Daily average forecasted air temperature T_ERA5_L (Top) and T_ERA5_MM (bottom) versus observed (T_Obs) values at representative sites for plain and mountain.

The ERA5_L reanalysis dataset exhibited high correlation with Tair values at most of the study stations (Fig. 2, Table. 2), with the R2 values ranging from 0.93 to 0.98. However, the PBIAS values varied widely, ranging from 0.04% at Agdal site to 87.17% at Neltner. Generally, PBIAS values are much higher in mountainous stations compared to plain sites, which is not surprising considering the low spatial resolution of ERA5_L and the strong relief. Moreover, the PBIAS values showed and underestimation of Tair by ERA5_L, except for the two higher stations (> 3000 m) where Tair is overestimated. The RMSE values varied from 0.98 to 1.76 °C in plain areas and from 2.11 to 4.84 °C at mountainous stations.

**Table 2:** The resulting daily performance by comparing agrometeorological estimates from the ERA5_L reanalysis dataset with ground observations; RMSE, R2, and PBIAS, denote root mean square error, correlation coefficient, and percent bias, respectively.

Stations	Air temperature (Tair)			Relative humidity (RH)			Wind speed (u2)			Solar radiation (Rs)		
	RMSE	R^2^	PBIAS	RMSE	R^2^	PBIAS	RMSE	R^2^	PBIAS	RMSE	R^2^	PBIAS
	°C		%			%	m s^−1^		%	Wm^−2^		%
Agdal	1.07	0.97	0.35	12.10	0.80	− 15.98	1.01	0.05	− 54.92	55.02	0.62	12.43
Chkhaoua	1.31	0.96	− 2.04	8.27	0.76	− 5.65	1.08	0.31	− 45.58	54.29	0.64	− 5.73
Graoua	179	0.97	− 14.19	4.78	0.82	5.34	0.47	0.28	− 51.18	51.52	0.61	− 0.57
R3	1.48	0.98	− 5.76	12.13	0.78	− 13.83	1.12	0.03	0.65	61.60	0.55	16.10
Ighzer	3.63	0.94	− 22.06	11.91	0.60	− 12.60	1.14	0.10	− 61.91	–	–	–
Irmker	4.30	0.92	28.92	9.74	0.6.1	3.78	–	–	–	–	–	–
Aremd	3.50	0.92	− 30.79	15.45	0 55	− M.32	0.59	0.36	− 46.75	62 55	0.58	16.67
Tachcdlrt	4.32	0 94	− 36.94	11.82	0.56	5.09	–	–	–	–	–	–
Netfner	4.84	0.96	87.18	14.13	0.44	5.52	–	–	–	–	–	–
Oukalmden	2.11	0.97	33.96	19.95	0.33	23.66	2.48	0.06	− 80.69	51.49	0.57	0.58

##### Tair MicroMet Vs in-situ measurements

Our results indicate that downscaling the temperature led to an improvement of biases at most of the studied sites especially in mountains, except for two higher stations for which the biases change but remain very high. R2 values remained generally unchanged (Fig. 2, Table. 3), and we observed lower RMSE as a consequence of bias improvement.

**Table 3:** The resulting daily performance by comparing agrometeorological estimates from the downscaled ERA5_L-MicroMet dataset with ground observations; RMSE, R2, and PBIAS, denote root mean square error, correlation coefficient, and percent bias, respectively.

Stations	Air temperature (Tair)			Relative humidity (RH)			Wind speed (u2)			Solar radiation (Rs)		
	RMSE	R^2^	PBIAS	RMSE	R^2^	PBIAS	RMSE	R^2^	PBIAS	RMSE	R^2^	PBIAS
	°C		%			%	m s^−1^		%	W m^−2^		%
Agdal	0.96	0.97	0.16	8.30	0.80	− 9.32	0.52	0.07	2.25	42.86	0.70	7.69
Chkhaoua	1.31	0.97	3.36	6.62	0.82	− 3.67	0.70	0.47	14.79	55.57	0.72	− 12.10
Graoua	0.93	0.97	− 5.21	5.09	0.82	7.69	0.26	0.51	21.52	160.68	0.68	− 64.43
R3	1.53	0.98	5.80	11.18	0.82	− 13.74	0.66	0.01	− 10.84	51.60	0.63	11.10
Ighzer	1.68	0.92	− 9.78	7.44	0.64	− 11.97	0.23	0.02	− 6.99	–	–	–
-Irmker	1.99	0.94	− 8.94	9.95	0.65	5.81	–	–	–	–	–	–
Aremd	2.50	0.85	1.14	11.83	0.63	− 5.73	0.89	0.43	84.45	139.9	0.59	− 62.29
Tachcdlrt	1.99	0.94	− 11.07	12.62	0.65	18.30	–	–	–	–	–	–
Netfner	3.47	0.91	− 51.47	18.59	0.42	27.82	–	–	–	–	–	–
Oukalmden	3.60	0.90	54.99	20.34	0.29	21.03	1.51	0.01	− 16.75	65.44	0.55	− 13.43

#### Relative humidity (RH)

##### RH ERA5_L Vs in-situ measurements

**Fig. 3:**
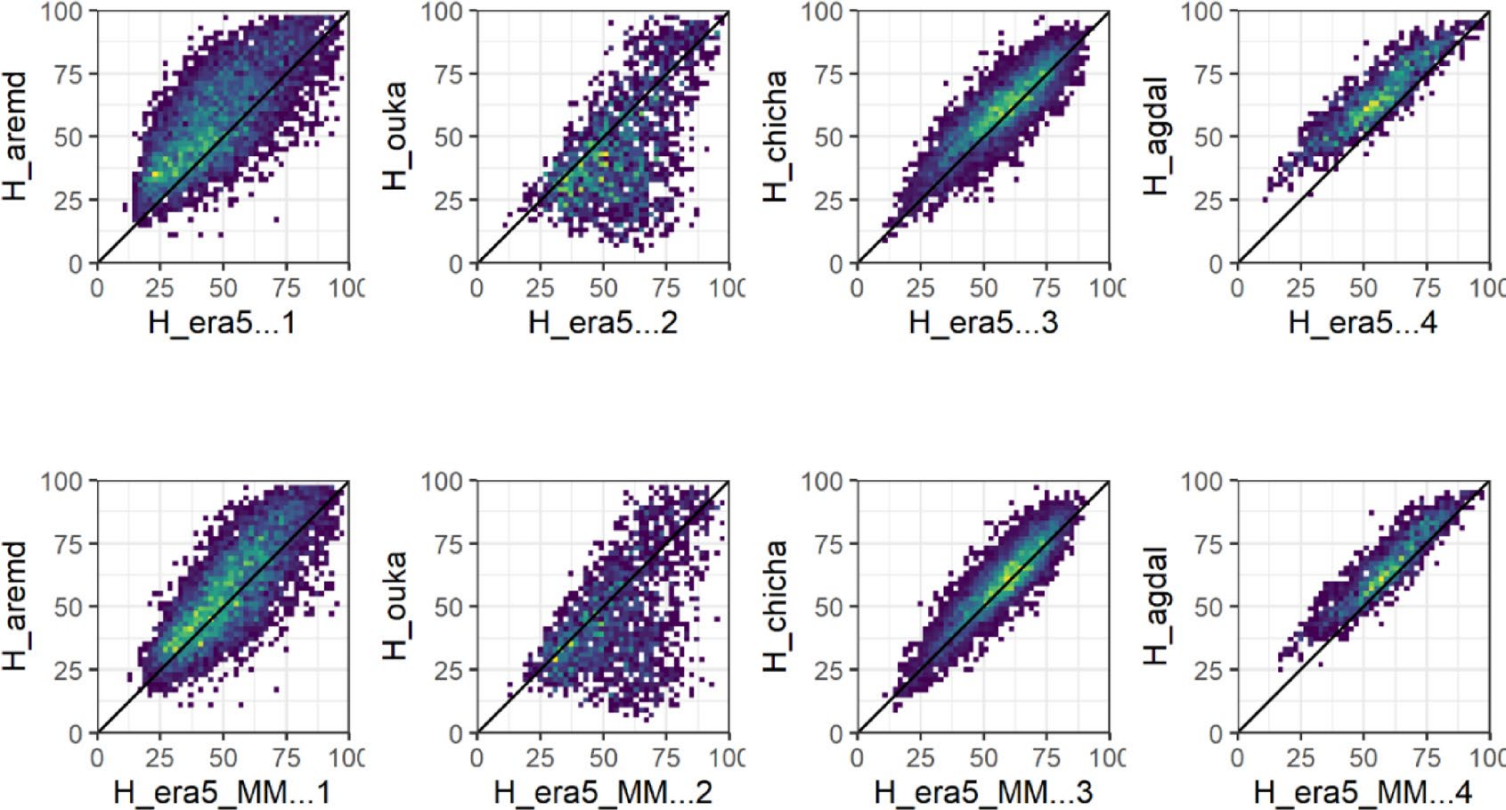
Daily average forecasted Relative humidity RH_ERA5_L (Top) and RH_ERA5_MM (bottom) versus observed (RH_Obs) values at representative sites for plain and mountain.

The ERA5_L relative humidity data were correlated with ground measurements, with R2 ranging from 0.33 to 0.86. Better correlation was observed for the plain sites (R2 > 0.75) compared to the mountainous sites (0.33 < R2 < 0.65), suggesting a topographic effect on ERA5 Land’s relative humidity estimates. PBIAS values ranged between -15.98% and 23.66%, showing an underestimation of ERA5_L’s relative humidity in plain sites, and an overestimation in mountain sites, except for Aremd station, which is in a valley between mountains. The RMSE varied between 4.78 and 19.3% (Fig. 3, and Table 2) and could be related to the topographic effect.

##### RH MicroMet Vs in-situ measurements

After downscaling, the RH showed relatively no significant improvements in most metrics, especially at the mountain stations. The majority of plain stations showed slight improvements in all metrics (Fig. 3 and Table 3).

#### Solar radiation (Rs)

##### Rs ERA5_L Vs in-situ measurements

**Fig. 4:**
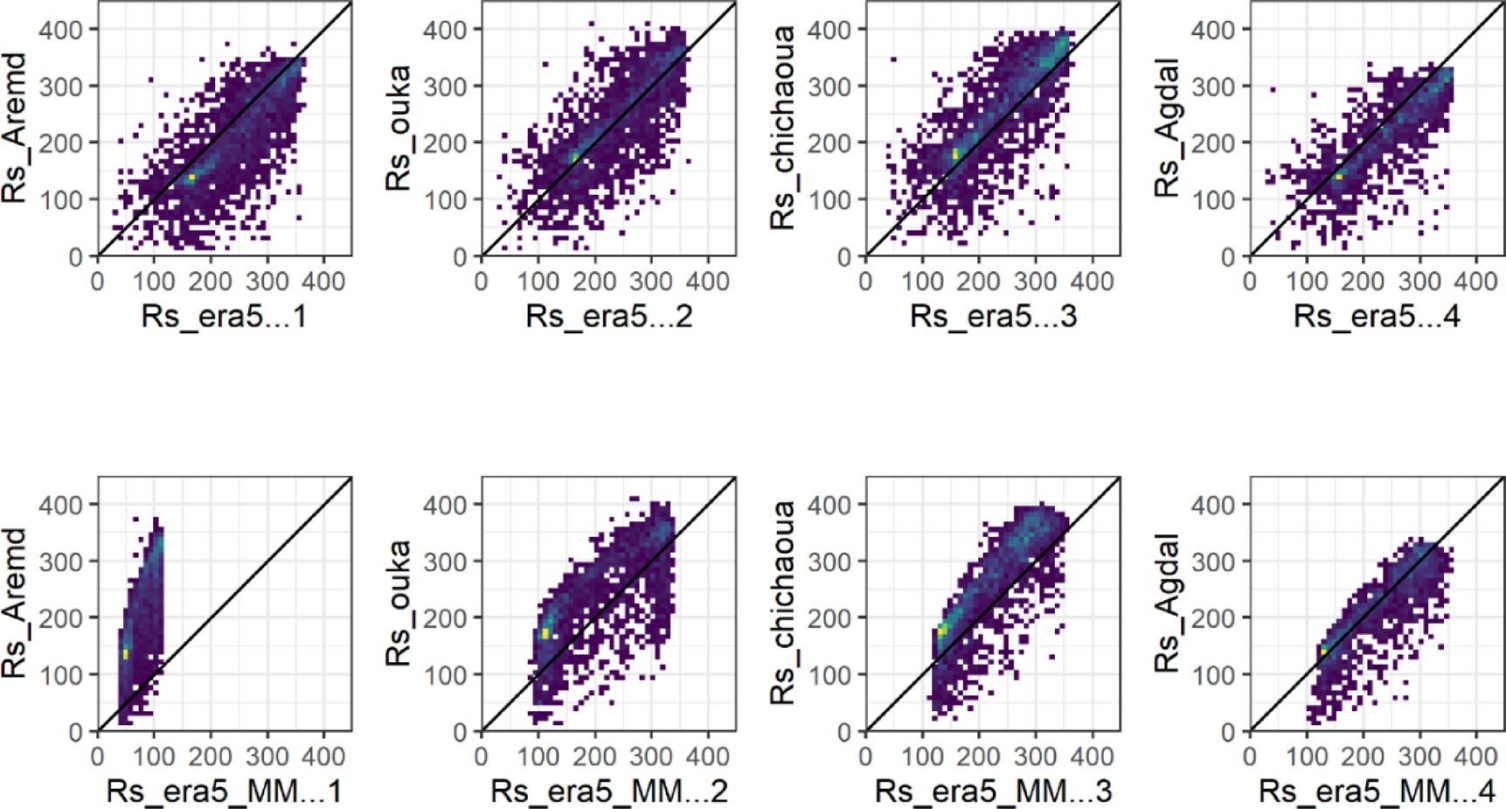
Daily average forecasted Solar radiation Rs_ERA5_L (Top) and Rs _ERA5_MM (bottom) versus observed (Rs _Obs) values at representative sites for plain and mountain.

The ERA5_L daily solar radiation estimation showed an acceptable correlation with observed data (0.57 < R2 < 0.69) and slight variations among stations. The PBIAS values varied from 0.58 to 16.67%. There was no clear effect of topography on Rs accuracy across all stations (Fig. 4, Table 2). The prediction of daily Rs values showed RMSE values between 51.49 W m-2 and 62.55 W m-2 under different conditions.

##### Rs MicroMet Vs in-situ measurements

For all sites except Agdal, we observe a degradation of Rs quality, with an increase of both RMSE and PBIAS. It is worth mentioning that not all stations had measurements of the Rs, which affects the representativeness of this variable in the study region, especially in the mountainous area (Fig. 4, Table 3). However, the results obtained in Aremd were worse than those obtained for Oukaimeden. This difference can be explained by the location of the stations. Oukaimeden is located on the summit of the mountain, while Aremd station is located in a valley. Indeed, valleys are more sensitive to shadow effects, particularly at certain times of day or under given microclimatic and topographic conditions.

#### Wind speed (u2)

##### u2 ERA5_L Vs in-situ measurements

**Fig. 5:**
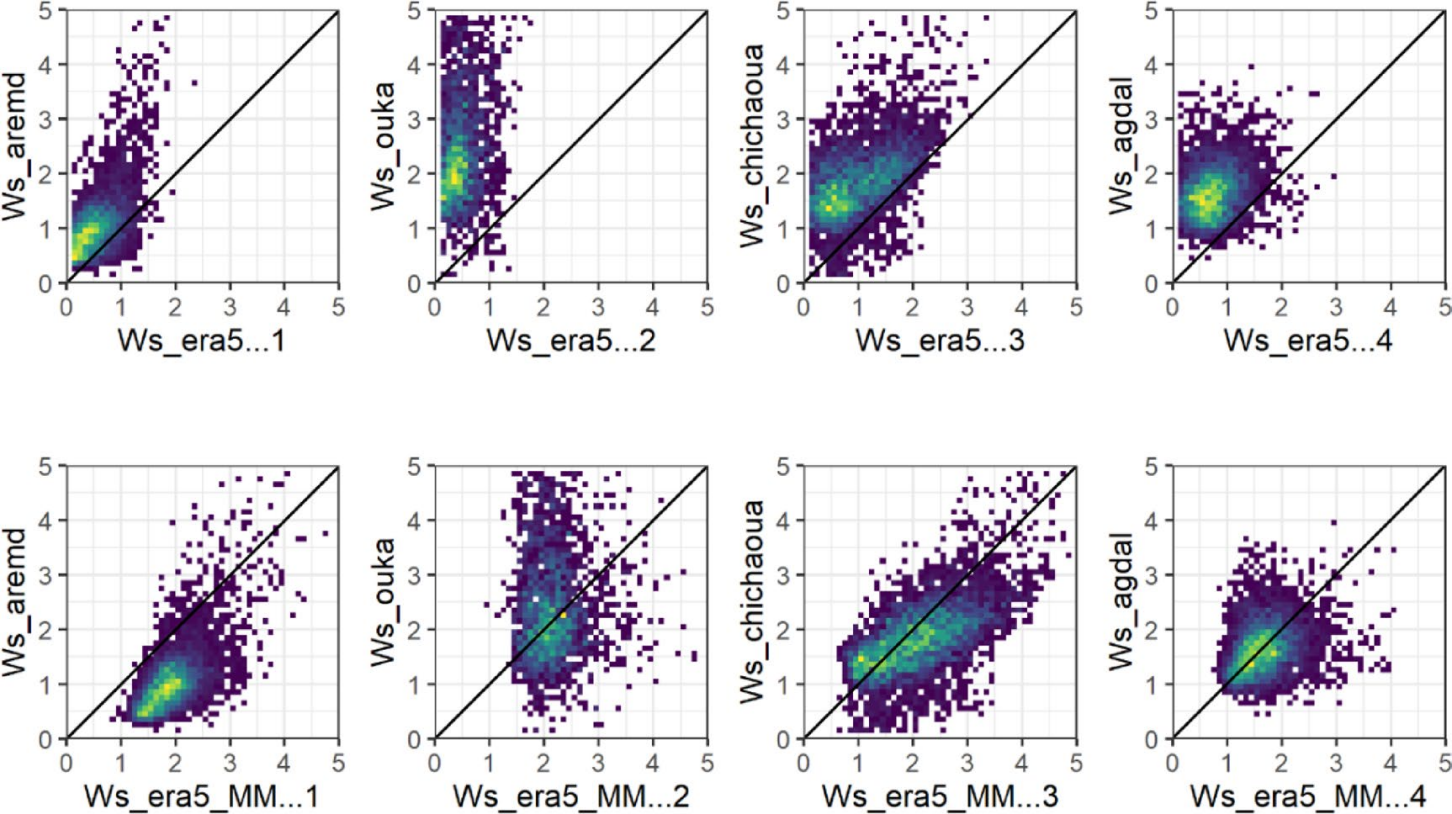
Daily average forecasted wind speed u2_ERA5_L (Top) and u2_ERA5_MM (bottom) versus observed (u2_Obs) values at representative sites for plain and mountain.

Regarding the daily Wind speed, our results demonstrate the poor accuracy and performance of the ERA5_L dataset in predicting u2 values. The R2 values are generally low (< 0.5) and highly variable, ranging from 0.03 to 0.36 (Fig. 5, Table 2). PBIAS values are generally high and vary from − 46.75 to − 80.69%, indicating an underestimation at all sites except Sidi Rahal where the PBIAS is low 0.65%. This poor accuracy of u2 could be related to the microclimate effects due to land cover and local topography, which leads to RMSE values ranging from 0.47 to 2.48 m s^−1^.

##### u2 MicroMet Vs in-situ measurements

The results of downscaling the u2 using MicroMet model were the best among all variables, showing relatively a significant improvement in the study area for both plain and mountain locations. Most stations showed a clear improvement in all metrics, except for Aremd where a slight rise in RMSE and PBIAS was noted. After disaggregation, PBIAS changed from underestimation to overestimation at all sites (Fig. 5, Table 3).

### Reference evapotranspiration (ET0)

#### ET0-ERA5_L Vs in-situ measurements

**Fig. 6:**
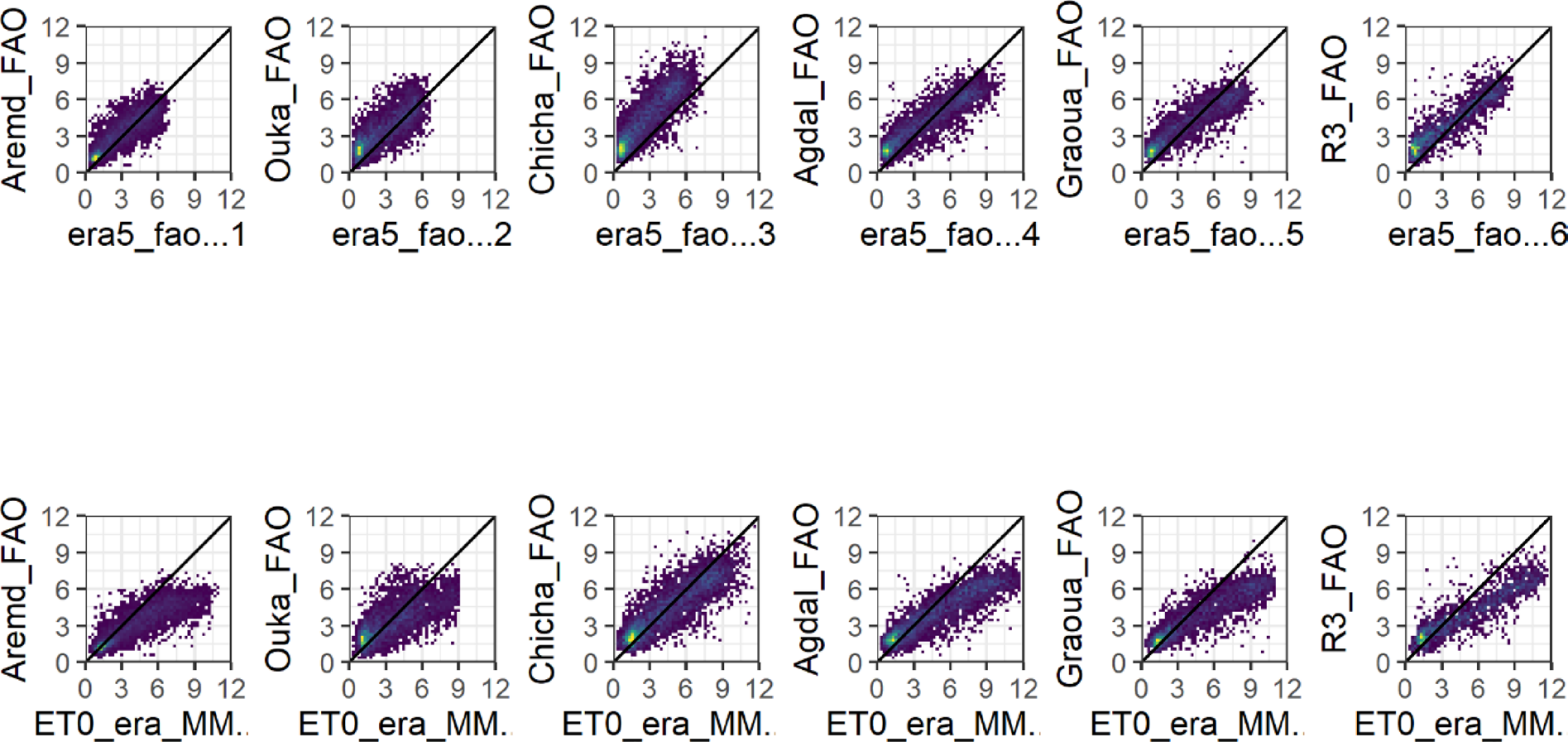
Daily average forecasted ET0_ERA5_L (Top) and ET0_ERA5_MM (bottom) versus observed (ET0_Obs) values at representative sites for plain and mountain.

ET0 estimates were performed for the study sites where all meteorological variables are available (six stations). The daily ET0 estimates using agrometeorological variables from the ERA5_L dataset showed R2 ranging between 0.6 and 0.8, PBIAS from − 37.06 to -2.02% and RMSE between 0.88 and 2 mm/day. The best performance was seen at the Aremd station (R2 of 0.72, PBIAS of − 7.08% and RMSE of 0.88 mm/day), while lower accuracy was found at the Chichaoua site (RMSE of 1.88 mm/day, R2 of 0.70, PBIAS of -37.20%). All sites showed negative PBIAS values, indicating that the ERA5_L dataset underestimates ET0 in both plain and mountainous sites, under different climate conditions over the Tensift basin (Fig. 6, Table 4).

**Table 4:** Comparison between ET0 computed using original ERA5_L and downscaled ERA5_L-MicroMet datasets with ground observations; Mean, RMSE, R2, and PBIAS, denote the mean ET0, root mean square error, correlation coefficient, and percent bias, respectively.

Stations	ETO_FAO_ERA5L (10 km)				ETO_FAO_MicroMet (250 m)			
	Mean	RMSE	R^2^	PBIAS	Mean	RMSE	R^2^	PBIAS
	mm/d	mm/d			mm/d	mm/d		
Agdal	4.11	1.20	0.78	− 2.02	5.21	1.48	0.79	28.59
Chichaoua	4.42	2.00	0.72	− 37.20	4.62	1.36	0.73	4.22
Graoua	3.77	1.01	0.78	− 2.37	4.94	1.52	0.78	27.80
Sidi Rahal	3.64	1.21	0.77	− 10.41	5.22	2.02	0.79	28.70
Aremd	2.84	0.88	0.72	− 7.08	4.16	1.71	0.70	36.2
Oukaimden	2.82	1.15	0.60	− 19.27	3.83	1.44	0.50	7.52

#### ET0-MicroMet Vs in-situ measurements

The calculated ET0 based on the downscaled ERA5_L variables showed generally quite different performance, but as a whole did not improve when compared with the original ERA5_L data. The R2 and RMSE remained relatively stable. However, the PBIAS increased for most of the study sites resulting in a shift from an underestimation of ET0-ERA5_L to an overestimation by ET0-MicroMet, except for Chichaoua and Oukaimeden (Fig. 6, Table 4).

Furthermore, in order to identify the main meteorological variables responsible for the changes in ET0 estimates, we performed a station-by-station analysis of ET0 and controlling variables. At Aremd, despite improvements in Tair and RH, the downscaling resulted in a strong overestimation of U2 and underestimation of Rs, resulting in ET0 slight overestimation. At Oukaimeden, the change from underestimation to slight overestimation was primarily linked to the overestimation of Tair and U2 after downscaling. In Chichaoua and Agdal, a slight overestimation of ET0 coincided mainly with the overestimation of U2. In Graoua, a slight increase of Tair and strong overestimation of U2 counterbalanced the strong underestimation of Rs and finally resulted in a slight overestimation of ET0. Finally, for R3, Tair increase was the dominant factor influencing the ET0 change, despite the underestimation of Rs (see Tables 2, 3, 4 and Fig. 6). In conclusion, original ERA5_L variable biases tend to partially offset each other when computing ET0, producing underestimation but reasonable correlations with observations. MicroMet downscaling slightly alters this balance; it generally increases U2 and Tair also degrading Rs performance in many cases. Overall, wind speed was the most influential variable responsible for overestimation of ET0 produced by Micromet. However, in many cases we see that, as for ERA5_L, Micromet variable biases compensate to produce an ET0 that was finally not that far from ground measurements, for example in Graoua.

Moreover, in this study model performance was evaluated using R^2^, RMSE, and PBIAS, which primarily serve as descriptive metrics. However, recent work by El Hachimi et al. (2023, 2025) provides a comprehensive sensitivity analysis of the FAO56-PM method, showing that reference evapotranspiration (ET₀) is most sensitive to air temperature (Tair), followed by solar radiation, wind speed, and relative humidity. Importantly, Tair is the most accurately represented variable in both the ERA5-L and AWS datasets and showed the greatest improvement after MicroMet downscaling. These findings suggest that, although a formal uncertainty propagation analysis was not conducted, the dominant driver of ET₀ is well constrained in our input data, thereby reducing the potential influence of input uncertainty on the ET₀ estimates.

### ET0 annual variability

**Fig. 7:**
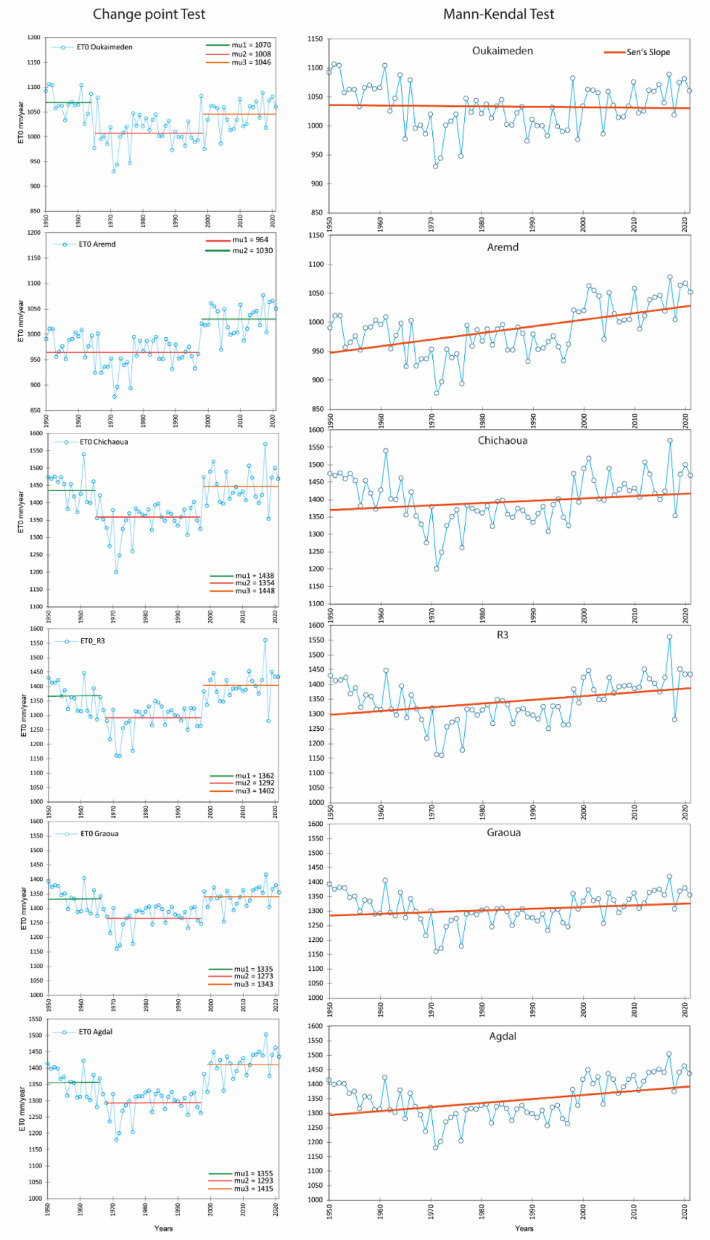
Change point and the Mann-Kendal trend tests of the annual ET0 from 1950 to 2021, where mu1, mu2, and mu3 are the annual mean ET0 (mm/year) before and after the change point respectively.

Despite the fact that ERA5_L generally underestimated ET0, it showed a good correlation with regard to ground-based ET0 as underlined by R2 between 0.6 and 0.8 at all the studied sites. So, we assumed that the same level of reliability would extend to the historical ERA5-Land data for the period 1950–2000. For this reason, and because MicroMet does not clearly improve these correlations for all the used variables (e.g., solar radiation), we decided to use the original ERA5_L dataset to investigate the spatio-temporal trend and dynamics of ET0 in the Tensift basin from 1950 to 2021. Figure 7 shows the evolution of annual ET0 for different locations in the Tensift basin. ET0 observed over the plain sites (Agdal, Graoua, R3, Chichaoua) showed the highest values, ranging from 1250 to 1500 mm/year, while mountainous stations (Aremd, Oukaimeden) showed lower ET0 values ranging from 1000 to 1100 mm/year. These lower ET0 values can be attributed to lower temperatures at higher altitudes, which limit evaporation rates^30^. Moreover, the evolution of annual ET0 can be divided into three main periods based on the change-point statistical test results. The first period, from 1950 to 1970, shows a downward trend in annual ET0. This was followed by a second period, from 1970 to 2000, during which annual ET0 remained relatively stable. The third period, from 2000 to 2021, was characterized by a significant increase in annual ET0 (Fig. 7). These findings apply to both plain and mountain stations. Even though there were three distinct phases in the evolution of annual ET0, the overall trend remained predominantly towards increase, as indicated by the positive trend observed in the Mann–Kendall test and the Sen’s slope.

To investigate the main factors influencing ET0 trends, we analyzed the four key climatic variables used to calculate ET0, at two representative stations: Chichaoua (plain) and Oukaimeden (mountain). Tair showed a slight decrease from 1950 to 1970, followed by a consistent increase from 1972 to the present, which was observed in both plain and mountain regions. In contrast, for the same periods there was an inverse evolution of RH. The changes of Rs and u2 were similar to those of ET0, with three distinct periods, a decrease 1950–1970, stability 1972–2000, and then a subsequent increase from 2000 to 2021. These observed changes result in three distinct periods of ET0 evolution. The first period, from 1950 to 1970, has been characterized by a cooling that can be considered as natural climatic variability, involving a slight decrease in Tair and Rs, and an overall increase in RH. The second period, from 1972 to 2000, displayed a variable evolution of all variables, with no clear downward or upward trend. The third phase, from 2000 to 2021, showed a significant increase in Tair, which emerged as the main driver of the ET0 increasing trend. This major increase in Tair could potentially be interpreted as the impact of climate change on the study area. Looking closely to the 2000–2021 period, the increase in ET0 is also sustained by an increase of u2, and to a lesser extent to an increase in Rs, particularly at Oukaimeden.

## Discussion

This study presents a reliability analysis of original and downscaled ERA5_L products in comparison with on-site meteorological variables (Tair, RH, Rs, and u2) from ten AWSs that were used in the ET0 calculation at six of these sites. We would remind that the grid point reanalysis data were compared to in situ observations data collected at AWS sites. Therefore, we cannot expect complete alignment between the two datasets, especially when working over complex topographic terrain. Yet, the results demonstrated an overall good agreement between ERA5_L products and the ground-based variables. Although Tair estimates exhibited the highest level of correlation among the reanalysis predictions, yet, Tair also showed an important underestimation, especially over mountainous stations, except for Neltner and Oukaimeden where Tair is overestimated. This could be explained as a topographic effect, considering the high difference of elevation that may occur between the station and the ERA5_L grid point with which it is compared. The RH performance was similar to Tair in terms of correlation, which can be related to the climatic conditions. The Rs showed an acceptable correlation with R2 around 0.6 at all sites, the Rs performance were similar at both plain and mountain stations. On the other hand, u2 yielded a poor correlation with ground data in addition to an underestimation at most studied sites; this inconsistency in u2 was noted by^25^ who suggested that it could be attributed to the omission of sea surface impact in ERA5_L product simulations. Furthermore^47^, highlighted that the inadequate performance of u2 could be explained by the fact that it resulted from a dynamic downscaling from ERA5. The overall results were consistent with previous studies conducted in North Mediterranean basins. For instance^33^, analyzed the accuracy of ERA5 and ERA5_L data for Italian irrigation districts. Their results showed patterns comparable to our study, particularly for u2. They outlined the highest accuracy for Tair estimates, followed by RH, Rs, and u2, for both reanalysis datasets, particularly under temperate climate conditions.

After the MicroMet disaggregation, Tair has been improved at most sites, excluding Oukaimeden, which may be due to the pronounced topographical effect that remains despite the corrections implemented in MicroMet. Most notably, u2 showed the utmost improvement across all the studied sites, and this may be associated with the fact that MicroMet takes into account land cover and topographical changes, which are important elements for u2. For RH we do not observe an improvement after MicroMet, the metrics remained relatively stable. Finally, for Rs, an overall degradation of biases was observed, which increase, particularly at mountainous sites, which could be linked to a poor consideration of topography. For example, the poor performance of Rs after disaggregation at Aremd could be due to the high spatial heterogeneity of slope orientation in the close neighborhood of this station.

Previous studies have indicated that the accuracy of reanalysis data can be influenced by terrain characteristics. Several investigations have examined the correlation between elevation and the accuracy of climate reanalysis data, specifically focusing on ERA-Interim^48^. Their findings demonstrated that disparities in altitude could have a significant impact on the accuracy of reanalysis data, particularly in regions with high elevations^49^. Consistent with these findings, our study also observed generally lower performance of ERA5_L evaluated variables in mountainous areas, including for the downscaled variables. The reanalysis datasets overall better reproduced the meteorological variables at altitudes below 1,000 m above sea level (m.a.s.l), which is in agreement with previous studies^33,50^.

**Fig. 8:**
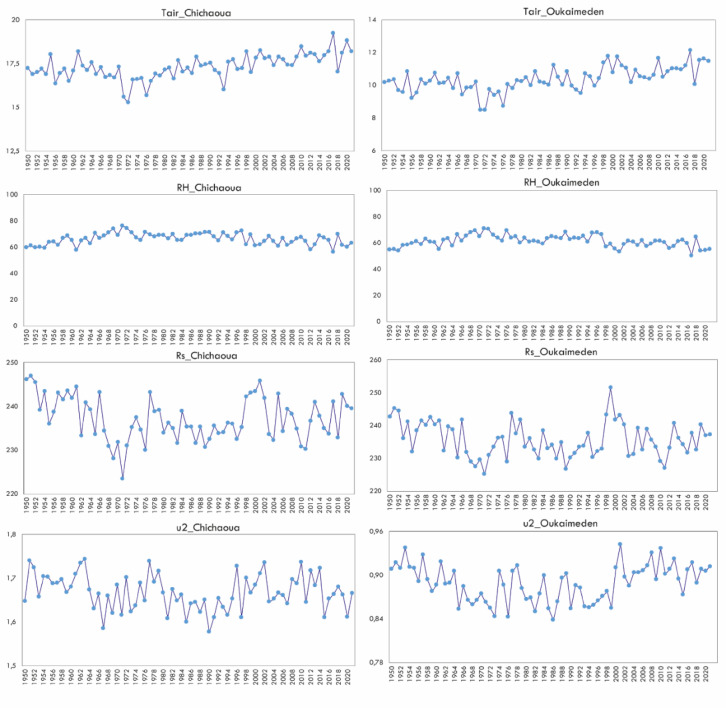
Annual evolution of the four climatic variables used to calculate ET0 at both plain (Chichaoua) and mountain (Oukaimeden) sites for the period between 1950 and 2021.

Despite the persistent bias after MicroMet, the downscaled ERA5_L data yielded nearly the same accuracy for ET0 estimates as compared with the original ERA5_L data, with an overestimation as shown by the PBIAS values (Table 4, Fig. 6). Several researchers have examined the sensitivity of ET0 to climatic variables. In a recent study^51,52^, used machine learning techniques to investigate a set of meteorological variables measured at a single station and identified the significant factors influencing ET0 performance in a semi-arid region of Morocco. Their results revealed that Tair and Rs had the most significant influence ET0 estimates, followed by RH and u2. In the same line^7^, reported that accurate ET0 estimates under Iranian conditions were mainly attributed to the good performance of Tair and u2. Similarly, several studies have examined the sensitivity of ET0 to meteorological variables on the Tibetan Plateau^53–55^. These studies indicated that u2 was a key factor controlling ET0 variation^53,56,57^, as well as u2, RH and Tair^58^, RH^55^, and Tair and u2^53^. In our study, we found that the good estimation of ET0 for Chichaoua was linked to the good performance of Tair, Rs, and u2 (Fig. 8), particularly after data disaggregation. Conversely, the increase in RMSE and PBIAS values for Rs and u2 at Graoua and Aremd stations may be the main factor contributing to the overestimation of ET0 at these sites. On the other hand, the low correlation of u2 with R2 of 0.01 at Oukaimeden and R3, and 0.07 at Agdal, combined with the slight overestimation of Rs, could be the main factor leading to the overestimation of ET0 at these sites.

Regarding the spatiotemporal evolution of ET0 for the period between 1950 and 2021, the ET0 changes showed a general increase over both plain and mountain areas in the Tensift basin. The 1950–1970 period reveals a declining tendency in annual ET0, which can be attributed to the fact that this period was identified as a wet phase, particularly in the 1950s and 1960s, with a few very wet years in the 1970s^59^. However, since 1980, the climatic variables have shown an increasing trend due to climate warming^60^, along with a decrease in precipitation leading to a succession of dry years^44^. Consequently, a significant increase in ET0 was observed during the dry period from 1980 to 2021, with the warmest years of the period recorded in 2010 and 2015^60^. Likewise^61^, highlighted the significant effect of climate change on temporal trends in ET0, resulting in an overall increase in annual ET0 in Egypt from the 1980s onwards. Similarly, a positive increase in annual ET0 has been reported over Spain between 1961 and 2011^62,63^, and in Greece based on 1983- 2001 period^64^. Both Mediterranean and North Africa regions repeatedly demonstrate these tendencies, implying that atmospheric evaporation demand may be increasing in response to the impacts of climate change. As a result, drought conditions in the region are likely to become more intense^61,62^.

Although the disaggregated product showed improvements in most analyzed variables except Rs, the performance in estimating ET0 was generally degraded as compared to that of the original ERA5_L product with a clear overestimation. Although ERA5_L accurately replicated ET0 observations in some sites, there is still important biases (over 10 to 20%) and they increase after MicroMet, which poses problems for agricultural application. Further analysis is required to understand these biases and try to improve the disaggregation methods. Thus, our results are less optimistic than previous studies like^65^ who investigated the global irrigation requirements of 26 crops using the Hargreaves-Samani method^66^ to calculate ET0 based on Tair and Rs information from the ERA5 dataset. Furthermore^33^, demonstrated that ERA5_L could be used as a substitute for ground measurements in estimating ET0 throughout Italy. However, since a coherent network of well-distributed climate stations is not available across both the plains and mountains of the African basins, reanalysis data sets stand as an option for simulating ET0 having in mind the limitations of the products.

## Conclusion

This study aimed to evaluate the performance of ERA5_L meteorological products in representing agrometeorological data over a 18-year period from 2003 to 2021 and to compare it with ground-based measurements at 10 sites located in both plain and mountainous regions at altitudes between 450 and 3200 m.a.s.l. within the Tensift basin in North Africa. The ERA5_L variables were downscaled to 250 m using MicroMet, and reference evapotranspiration ET0 was computed using FAO56-PM at the studied sites using both the original and downscaled variables.

Our results showed that the daily average Tair estimates from ERA5_L were the most accurate reanalysis predictions, followed by Rs, RH, and u2, leading to an overall reliability in daily ET0 estimates despite some noticeable exceptions. Regarding the downscaled data, Tair and u2 clearly improved, no improvement was observed for RH, and a degradation of Rs was obtained with an increase of biases. Therefore, statistical metrics for ET0-MicroMet estimates were degraded as compared to ET0-ERA5_L, with an overestimation highlighted by PBIAS values.

Despite these limitations, ERA5_L dataset may be a useful data source to estimate ET0, especially in plain areas when not enough ground data is available. However, regarding the u2 variable, which showed the most significant improvement after disaggregation, it is recommended to use MicroMet as a downscaling tool for studies requiring this particular variable.

Lastly, the study investigated the spatio-temporal changes in ET0 across the Tensift basin over the period 1950–2021. ET0 trends showed three main phases, where annual ET0 decreased between 1950 and 1970, remained nearly stable in 1970–2000, and then underwent a significant increase from 2000 to 2021. While the 1950 to 1970 can be considered as representing natural climate fluctuations, the period 1972–2021 was mainly influenced by changes in Tair, highlighting the effect of climate change on ET0 trends.

These findings contribute to our understanding of the reanalysis data reliability under various climatic and topographical conditions and their optimal use. This study also provides information for water managers and stakeholders to use the reanalysis data as an alternative data source in estimating ET0 for better water management in North African basins, where the availability of agrometeorological data is limited.

## Data Availability

The datasets generated and/or analyzed during the current study are not publicly available but can be provided by the corresponding author on request.
